# A High Performance Load Balance Strategy for Real-Time Multicore Systems

**DOI:** 10.1155/2014/101529

**Published:** 2014-04-14

**Authors:** Keng-Mao Cho, Chun-Wei Tsai, Yi-Shiuan Chiu, Chu-Sing Yang

**Affiliations:** ^1^Institute of Computer and Communication Engineering, Department of Electrical Engineering, National Cheng Kung University, Tainan 70101, Taiwan; ^2^Department of Applied Informatics and Multimedia, Chia Nan University of Pharmacy & Science, Tainan 71710, Taiwan

## Abstract

Finding ways to distribute workloads to each processor core and efficiently reduce power consumption is of vital importance, especially for real-time systems. In this paper, a novel scheduling algorithm is proposed for real-time multicore systems to balance the computation loads and save power. The developed algorithm simultaneously considers multiple criteria, a novel factor, and task deadline, and is called power and deadline-aware multicore scheduling (PDAMS). Experiment results show that the proposed algorithm can greatly reduce energy consumption by up to 54.2% and the deadline times missed, as compared to the other scheduling algorithms outlined in this paper.

## 1. Introduction

To promote convenience in people's lives, “smart” has become a new requirement for various products [1], which in turn has led embedded systems to being developed. Embedded systems are now widely used in our daily life, such as digital appliances, network devices, portable devices, and diversified information products [2–8]. Various applications are employed in these devices, and multimedia applications are especially prevalent [9–11]. In order to support the plethora of applications, particularly multimedia-related signal processing, superior performance of embedded systems is required. Along with the increasing demand, the system energy consumption is also increasing. As a matter of fact, the advancement in battery technologies has been slower than the advancement of computing speed and the consequent processor energy consumption.

Due to these reasons and to enhance the performance of modern embedded systems [12–14], the system needs to (1) provide more computation power and (2) reduce power consumption while maintaining performance.

To enhance the* performance* of the embedded system, a multicore architecture is one of the possible solutions which allow the system to process numerous jobs simultaneously by parallel computation. Keeping every processor core of the system in high utilization is an important issue to achieve high performance. In order to maximize the parallel computing of a multicore system, load balance becomes an issue that needs to be considered when scheduling. Round robin is one of the simple methods to dispatch tasks in a multicore system [15], where tasks are dispatched to processor cores in a rotated order. Shortest queue first [16] is another method that is often used. In this method, tasks are assigned to the processor core with the shortest waiting queue. To find the shortest queue, the number of tasks or the total computation time of tasks on the processor core can be used to represent the queue length. The latter is also called shortest response time first, and requires a priori knowledge about task service times. Additionally, utilization of a processor is usually considered as the criterion in load balance. To generate the maximum balanced load, tasks should be assigned to the processor core with the lowest utilization [17].

In addition to the performance of the embedded system, energy consumption is also an important issue. Over the last decade, manufacturers have been competing to improve the performance of processors by raising the clock frequency. Under the same technology level and manufacturing processes, the higher operating frequencies of a CMOS-based processor require higher supply voltage. The dynamic power consumption (*P*_dynamic_) of a CMOS-based processor is related to the operating frequency (*f*) and supply voltage (*V*_dd_) as *P*_dynamic_ ∝ *V*_dd_^2^ × *f*. Thus, higher operating frequency results not only in higher performance but also higher power consumption. Due to the fact that devices which use batteries carry limited energy, research on power saving has received increasing attention, where dynamic voltage and frequency scaling (DVFS) techniques are often applied to extend the battery life of portable devices. DVFS reduces the supply voltage and operating frequency of processors simultaneously to save energy when performance demand is low. Just as the human brain consumes a lot of energy, the processors of a system consume the majority of the energy too. Consequently, multicore architectures can benefit greatly from DVFS technology. In early multicore systems, all processor cores shared the same clock [18]. Under this architecture, DVFS can still be applied to save energy, but there are more limitations. The tradeoff between performance and energy consumption becomes more difficult. To support more flexible power management for multicore systems, the voltage and frequency island (VFI) technique [19, 20] has been developed, where processor cores are partitioned into groups, with processor cores belonging to the same group sharing one supply voltage and having the same processing frequency [21].

### 1.1. Motivation

In the past, most studies regarding scheduling on a multicore system [22–25] have not been designed for real-time systems. For some urgent tasks, raising the priority level of these tasks cannot satisfy the urgency completely. In this case, not only a priority but also a deadline will be used to express the character of this task. Tasks with deadlines are called real-time tasks. Nowadays, some studies have focused on scheduling for real-time multicore systems [26, 27]. However, these kinds of algorithms usually view guaranteeing the hard deadline as their main purpose, and therefore limitations arise. Also, these algorithms need more a priori knowledge of tasks. When implementing them into a real system, they must satisfy some requirements, such as fixed application, training, and specific information about the application. However, most portable devices execute nonspecific applications, which are usually not hard-real-time tasks. For example, users may download numerous applications onto their smart phones, where most are soft-real-time tasks and normal tasks. Unfortunately, it is difficult to ensure which applications will be executed on devices before users actually use them. This is why the design of algorithms for specific applications is not suitable, and the requirement of additional priori knowledge is difficult to implement efficiently. Thus, to solve these problems and to consider the tradeoff between performance and energy consumption, this paper applies a solution to the problems of scheduling and power saving in a real-time system for a multicore platform. The proposed algorithms decrease the times of deadline missed and simultaneously consider dynamic power, static power, load balance, and practicability.

### 1.2. Contribution

The contributions of this paper are as follows.

1. A power and deadline-aware multicore scheduling algorithm is proposed. It is composed of two parts: a power-aware scheduling algorithm and a deadline-aware load dispatch algorithm. The proposed algorithm is simple and easy to implement and overcomes the problems related to many existing power-saving algorithms that are difficult to implement and not suitable for diverse applications.
2. In the frequency-scaling part of the power-aware scheduling algorithm, we propose a DVFS-based algorithm called ED^3^VFS. This algorithm uses task deadlines to determine when to scale the operating frequency and is able to adjust parameters dynamically to suit different task sets. Experimental results show that ED^3^VFS is very effective and flexible.
3. This paper also proposes a deadline-aware load dispatch algorithm, called the two-level deadline-aware hybrid load balancer. The proposed load dispatch algorithm includes two levels: the concept of load imbalance in the first level and a novel load balance strategy, distribution of task deadline, in the second level. We also combined the other load balance strategies in the second level and let the proposed load dispatch algorithm deal with real-time tasks and normal tasks simultaneously.
4. We implemented the proposed load dispatch algorithm in Linux and ported the MicroC/OS-II real-time operating system kernel to a PACDSP on a PAC Duo platform and implemented the proposed power-aware scheduling algorithm in the real-time kernel. Experimental results show that the proposed algorithms work well in a real environment.

### 1.3. Organization

The remainder of this paper is organized as follows.  Section 2 gives a brief introduction to work related to scheduling in a multicore system.  Section 3 discusses and defines the problems we aim to solve in this paper, as well as limitations and assumptions.  Section 4 describes the proposed power and deadline-aware multicore scheduling algorithm. A performance evaluation of the proposed algorithm is presented in Section 5, with conclusion offered in Section 6.

## 2. Related Work

### 2.1. DVFS-Based Power Saving Technologies

**Figure 1:**
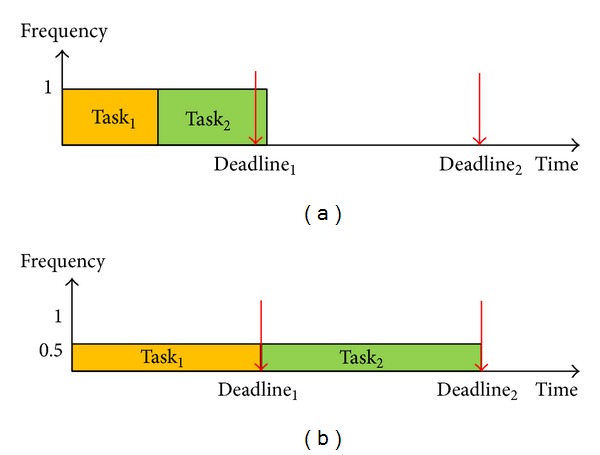
Scaling voltage and frequency at task slack time. (a) Without DVFS and (b) with DVFS.

There are two strategies for using DVFS techniques to reduce energy consumption. The first strategy is scaling voltage and frequency at task slack time. When a processor serves a task, the operating frequency is multiplied by the rate between the worst-case execution time (WCET) and the deadline of the task [28] to reduce power consumption, as shown in Figure 1. Shin et al. [29] combined offline and online components to satisfy the time constraints and reduce energy consumption. The offline component finds the lowest possible processor speed that satisfies all the time constraints, while the online component varies the operating speed dynamically to save more power. Since the task execution time may be changed slightly when executed, Salehi et al. [30] used an adaptive frequency update interval to follow sudden workload changes. The history data is used to predict the next workload and then, according to the prediction error, adjust the frequency update interval.

**Figure 2:**
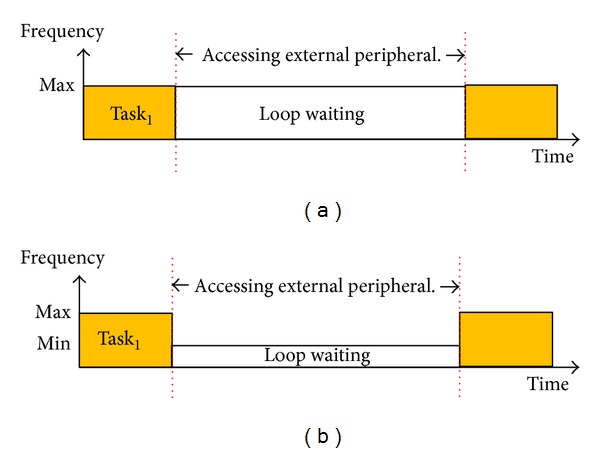
Scaling voltage and frequency when access external peripheral. (a) Without DVFS and (b) with DVFS.

The second strategy is scaling voltage and frequency when accessing external peripherals. References [31, 32] pointed out that the operating speed of memory and peripherals is much lower than that of the processors. For tasks that are memory-bounded or I/O-bounded, the operating frequency of a processor can be decreased to save power while waiting for the external peripherals to finish their jobs, as shown in Figure 2. Liang et al. [33] proposed an approximation equation called the memory access rate-critical speed equation (MAR-CSE) and then defined and used the memory access rate (MAR) to predict its critical speed.

### 2.2. Scheduling on Real-Time Multicore Systems

Because the classical approaches need a priori knowledge of the application to achieve the target, especially when real-time guarantees are provided, Lombardi et al. [34] developed a precedence constraint posting-based offline scheduler for uncertain task durations. This method uses the average duration of a task to replace the probability distribution and calculates an approximate completion time by this cheaper-to-obtain information. Kim et al. [35] presented two pipeline time balancing schemes, namely, workload-aware task scheduling (WATS) and applied database size control (ADSC). Because the execution time of each pipeline stage will change along with the input data, different execution times in each pipeline stage reduce the performance of a system. To achieve higher performance, the pipeline time of each pipeline stage must be in a balanced state. The basic idea of the pipeline time balance schemes is monitoring and modifying the parameter value of the function in each pipeline stage, thereby allowing the execution time of each pipeline stage to be close to the same average value. Jia et al. [36] presented a novel static mapping technique that maps a real-time application onto a multiprocessor system, which optimizes processor usage efficiency. The proposed mapping approach is composed of two algorithms: task scheduling and cluster assignment. In task scheduling, the tasks are scheduled into a set of virtual processors. Tasks that are assigned to the same virtual processors share the maximized data, while data shared among virtual processors is minimized. The goal of cluster assignment is to assign virtual processors to real processors so that the overall memory access cost is minimized.

In addition to balancing the utilization of each processor core, how to tackle the communications among tasks with performance requirements and precedence constraints is another challenge in the scheduling on real-time multicore systems. Hsiu et al. [37] considered the problem of scheduling real-time tasks with precedence constraints in multilayer bus systems and minimized the communication cost. They solved this problem via a dynamic-programming approach. First, they proposed a polynomial-time optimal algorithm for a restricted case, where one multilayer bus and the unit execution time and communication time are considered. The result was then extended as a pseudopolynomial-time optimal algorithm to consider multiple multilayer buses. To consider transition overhead and design for applications with loops, Shao et al. [38] proposed a real-time loop scheduling-algorithm called dynamic voltage loop scheduling (DVLS). In DVLS, the authors succeeded in repeatedly regrouping a loop based on rotation scheduling and decreased the energy consumed by DVS within a timing constraint.

In addition to the abovementioned studies, there are many research directions and issues regarding real-time multicore systems. For real-time applications, it is common to estimate the worst case performance early in the design process without actual hardware implementation. It is a challenge to obtain the upper bound on the worst case response time considering practical issues such as multitask applications with different task periods, precedence relations, and variable execution times. Yet, Yang et al. [39] proposed an analysis technique based on mixed integer linear programming to estimate the worst case performance of each task in a nonpreemptive multitask application on a multiprocessor system. Seo et al. [26] tackled the problem of reducing power consumption in a periodic real-time system using DVS on a multicore processor. The processor was assumed to have the limitation that all cores must run at the same performance level. And so to reduce the dynamic power, they proposed a dynamic repartitioning algorithm. The algorithm dynamically balances the task loads of multiple cores to optimize power consumption during execution. Further, they proposed a dynamic core scaling algorithm, which adjusts the number of active cores to reduce leakage power consumption under low load conditions.

Cui and Maskell [40] proposed a look-up table-based event-driven thermal estimation method. Fast event driven thermal estimation is based upon a thermal map, which is updated only when a high level event occurs. They developed a predictive future thermal map and proposed several predictive task allocation policies based on it. Differing from the utilization-based policy, they used the thermal-aware policies to reduce the peak temperature and average temperature of a system. Han et al. [27] presented synchronization-aware energy management schemes for a set of periodic real-time tasks that accesses shared resources. The mapping approach allocates tasks accessing the same resources to the same core to effectively reduce synchronization overhead. They also proposed a set of synchronization-aware slack management policies that can appropriately reclaim, preserve, release, and steal slack at runtime to slow down the execution of tasks and save more energy. Chen et al. [41] explored the online real-time task scheduling problem in heterogeneous multicore systems and considered tasks with precedence constraints and nonpreemptive task execution. In their assumption, the processor and the coprocessor have a master-slave relationship. Each task will first be executed on the processor and then dispatched to the coprocessor. During online operation, each task is tested by admission control, which ensures the schedulability. Since the coprocessor is nonpreemptive, to deal with the problem of a task having too large a blocking time, the authors inserted the preemptive points to configure the task blocking time and context switch overhead in the coprocessor.

### 2.3. Summary

To extend the system lifetime for energy-limited devices, one of the possible ways is to use DVFS-based technology [28, 29, 31, 32] to save energy. Because the requirements will change when the real-time system is being used, other studies [27, 42, 43] have combined DVFS technology and a real-time scheduler to meet the time constraint while reducing energy consumption. Now, under the environment of multicore architectures, researchers have proposed multicore schedulers, which can meet real-time constraints and consume lower energy. Along with the development of technology, the algorithms have become much more complex and have increasing restrictions when multiple issues need to be considered simultaneously. As a consequence, these algorithms become difficult to implement and work in real environments. Thus, this paper relaxed some limitations to allow the proposed algorithm to be easier to implement and work well in real environments with simultaneous consideration to the real-time, power, and load balance issues.

## 3. Problem Definition

**Figure 3:**
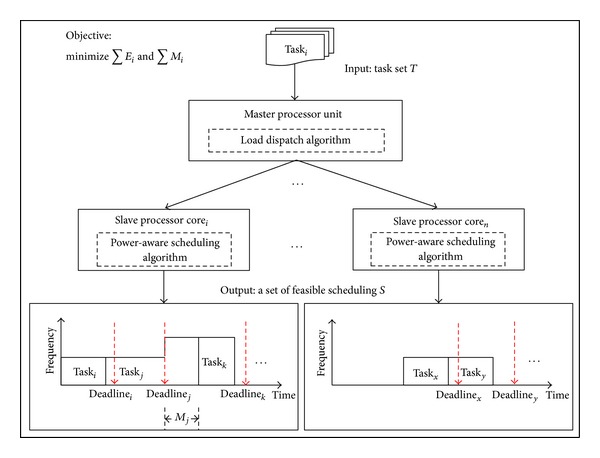
System model.

The DVFS-based power-aware scheduling problem is defined as finding a schedule that can satisfy all the constraints of a system while consuming less energy to execute tasks. Differing from the aforementioned traditional scheduling on a single core system, scheduling on a multi-core system needs to decide not only the execution order of tasks, but also which tasks should be executed on which processor core. A good load dispatch can improve the performance and reduce energy consumption, so load dispatch is a very important issue in multicore scheduling. In this paper, we divided the power-aware multicore scheduling problem into load dispatch and power-aware scheduling and proposed different algorithms to solve them individually. Additionally, the key points of using the DVFS technique to reduce the energy consumption of a system include deciding when the operating voltage and frequency should be adjusted and selecting the operating state. To relax the limitations and make the proposed algorithm easier to implement and more light-weight, missing deadline is allowed in this research. The problem we consider in this paper can be defined as follows and is illustrated in Figure 3.

*System Model*. There is one master processor unit and *n* slave processor cores in the system, and each processor core has its own operating system and can scale the operating voltage and frequency independently. When tasks are released, the master processor unit exchanges the status information with each slave processor core by IPC and dispatches them to a suitable slave processor core individually; then, the slave processor cores schedule tasks that are dispatched to them individually. Under this architecture, the proposed algorithm can apply to either homogeneous multicore systems or heterogeneous multicore systems and each processor core can manage itself. In this work, the platform that is used contains an ARM core as master processor unit and two DSPs.

*Input*. A task set *T* = {*T*_real_, *T*_normal_}, where *T*_real_ is the set of real-time tasks and *T*_normal_ is the set of normal tasks. Each real-time task can be represented as (*Release*_*time*, *Priority*, *Related*_*Deadline*) and it can be a periodic task or an aperiodic task. In a dynamic environment, it is difficult to get all the information about tasks and the overhead of using optimization method is too heavy when every task is released. Therefore, we tried to schedule real-time tasks without considering the execution time of tasks. Although hard deadline is not guaranteed, methods are proposed to decrease the missing-deadline probability. For normal tasks, we only use (*Release*_*time*, *Priority*) to represent them. Generally, the execution order of real-time tasks is based on the absolute deadline of tasks. *Priority* of real-time tasks is used only when two or more absolute deadlines are identical. The details of scheduling algorithm will be described in Section 4.

*Output*. A set of feasible scheduling, *S* = {*s*_1_, *s*_2_,…, *s*_*n*_}, where *s*_*i*_ is the scheduling result of the *i*th slave processor core and scaling operating voltage and frequency produced by the proposed scaling algorithm.

*Objective*. To minimize total energy consumption *E*_total_, the objective function is expressed in

(1) $$\begin{array}{c} \text{Minimize}\left(E_{\text{total}}\right), \end{array}$$

(2) $$\begin{array}{c} E_{\text{total}}=\sum E_{i}, \end{array}$$

(3) $$\begin{array}{c} E_{i}=\sum E_{ij}, \end{array}$$

where *E*_*i*_ is the energy consumption of the *i*th slave processor core and *E*_*ij*_ is the energy consumption of the *j*th task in the *i*th slave processor core, as expressed in

(4) $$\begin{array}{c} E_{ij}=\sum \left(P_{ijk}\times t_{ijk}\right), \end{array}$$

(5) $$\begin{array}{c} P_{ijk}=c\times V_{ijk}^{2}\times f_{ijk}, \end{array}$$

where *P*_*ijk*_ is the power of the *j*th task at the *k*th time slice in the *i*th slave processor core, expressed as (5), and *t*_*ijk*_ is the duration of the *k*th time slice for the *j*th task in the *i*th slave processor core. Since the operating mode will not always be the same for a given task under the proposed algorithm, the energy in each time slice must be individually calculated and then summarized. In (5), *c* is the load capacity, *V*_*ijk*_ is the operating voltage, and *f*_*ijk*_ is the operating frequency of the *j*th task at the *k*th time slice in* i*th slave processor core.

It is allowed that tasks are finished after their deadlines in this paper. The processing speed will be increased when a deadline is missed so that the task can be finished faster. Thus, the performance constraint can be expressed as

(6) $$\begin{array}{c} \text{Minimize}\left(\sum M_{i}\right), \end{array}$$

where *M*_*i*_ represents if the deadline of *i*th task is missed and is defined as

(7) $$\begin{array}{c} M_{i}=\{\begin{array}{cc} 1, & \;\text{if}\;\;ft_{i}>d_{i}\;\\ 0 & \;\text{otherwise}, \end{array} \end{array}$$

where *ft*_*i*_ is the finish time and *d*_*i*_ is the absolute deadline of the *i*th task.

## 4. Power and Deadline-Aware Multicore Scheduling

In this section, an efficient multicore scheduling algorithm is presented, called power and deadline-aware multicore scheduling, which integrates three different parts (modules): (1) mixed-earliest deadline first (MEDF) [42], (2) enhanced deadline-driven dynamic voltage and frequency scaling (ED^3^VFS), and (3) two-level deadline-aware hybrid load balancer (TLDHLB). Among them, MEDF is used to schedule the tasks that have been dispatched to a processor core. ED^3^VFS is an enhanced version of D^3^VFS [42] and is used to scale the operating mode on each slave processor core. And finally, TLDHLB is used for task dispatch and is composed of two levels: the first level is the load imbalance strategy, while the second level is load balance. For example, when a new task that needs to be served by DSP arrived, TLDHB will dispatch it to a DSP with consideration of load balance. After the DSP receives the task, MEDF will schedule the new task. At the same time, ED^3^VFS will be executed on DSP periodically to reduce the energy consumption.

### 4.1. Mixed-Earliest Deadline First

The original scheduling algorithm used in the MicroC/OS-II kernel is a fundamental priority-based scheduling algorithm. To support real-time tasks and normal tasks in the same time, mixed-earliest deadline first is selected to replace the original scheduling algorithm. MEDF combined EDF and fixed-priority scheduling. For real-time tasks, it uses EDF to schedule the tasks. When there are two or more deadlines of real-time tasks that are identical or for normal tasks, MEDF uses fixed-priority scheduling to decide the execution order. Moreover, MEDF will always select real-time tasks first when there are real-time tasks and normal tasks in the ready queue simultaneously.

To cooperate with TLDHLB to save static power, we modified MEDF to let it turn off the processor cores while the ready task, which has the highest priority, is idle while there is no real-time task or other normal tasks. This means that when a processor core finishes all tasks, it will turn itself off to save power.

### 4.2. Enhanced Deadline-Driven Dynamic Voltage and Frequency Scaling

The D^3^VFS scales operating mode dynamically by the active status of a system. *α* and *β* are two parameters used in D^3^VFS and are set as 10. D^3^VFS will scale operating modes while the system continues to be busy for *α* time units or when no deadlines are missed for *β* time units. Inspired by observations of D^3^VFS, we present a better strategy to set parameters *α* and *β* to enhance the performance of the scheduling system. First, in D^3^VFS, the related deadlines of tasks are not needed to be longer than 10 or a fixed threshold. When the shortest related deadline is shorter than this value of 10 (threshold), *α* and *β* become negative, which should be amended. Second, the purports of *α* and *β* are not exactly the same, and so their impacts are different. The bigger value we set to *α*, which is the longer time that the processor stays in lower speed, because the system will increase the operating frequency in a slower rate. This leads to more power savings with worse computing capacity. On the other hand, *β* is used for decreasing the operating frequency. A bigger *β* will allow the system to stay in high speed for longer, so that the performance will be better, but more power is consumed. Third, there is more than one task working in a system simultaneously. And in real environment, these tasks may not always be the same. The best setting for each task is different, and so giving different settings for different task sets is more flexible.

To solve these aforementioned problems, this paper proposes a different concept to improve the performance of power saving algorithms and conforms to real situations while the system is working.  Pseudocode 1 shows the pseudocode of ED^3^VFS. The basic idea of ED^3^VFS is that the settings of *α* and *β* are free to change along with different task sets and these two parameters will be set to two different values. According to the basic idea of ED^3^VFS, we ran a series of experiments to find a better setting and to verify that the new setting is superior to the original D^3^VFS setting. The experimental settings include two main groups and are described as follow.

- The first group is just like the original D^3^VFS, and the settings of *α* and *β* are the same. There are four settings in this group, and the four settings are *α* = *β* = *D*_*sr*⁡_ × 0.2, 0.4, 0.6, and 0.8, where *D*_*sr*⁡_ is the shortest related deadline.
- The second group used in ED^3^VFS also features four settings in. In this group, *α* and *β* will be set into different values. *α* = *D*_*sr*⁡_ − *β* and *β* = *D*_*sr*⁡_ × 0.8, 0.6, 0.4, and 0.2. Just as in the above, the effects of *α* and *β* are opposite, so we gave them opposite values.  Table 1 lists the overall settings of *α* and *β* in these experiment series.

**Figure 4:**
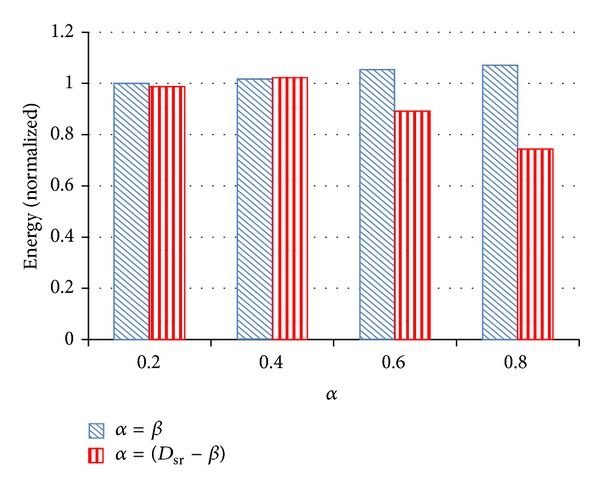
Energy comparison between original D^3^VFS and ED^3^VFS.

**Figure 5:**
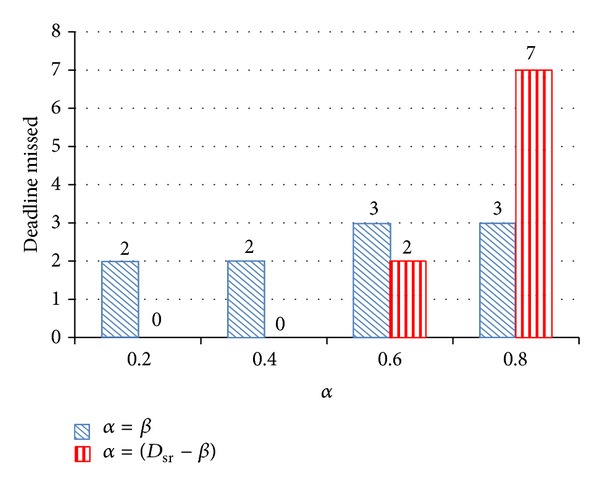
Performance comparison between original D^3^VFS and ED^3^VFS.

Figure 4 shows the energy comparison between the two different settings, namely, the original D^3^VFS and ED^3^VFS. The vertical axis shows energy consumption while the horizontal axis shows the setting of *α*, where *α* = *D*_*sr*⁡_ × 0.2,  0.4,  0.6, and 0.8. In this research, the first experimental result of energy is used to normalize all of the other results and make them easy to see the differences in comparison. The results show that the energy consumption of ED^3^VFS is lower than the original D^3^VFS in most cases.  Figure 5 shows the performance comparison between original D^3^VFS and ED^3^VFS. We used the number of deadlines missed as the criterion to compare their performance. For a real-time system, a lower number of missed deadlines are better. The results show that ED^3^VFS is better than the original D^3^VFS in performance, except for the case when *α* = *D*_*sr*⁡_ × 0.8. Although the proposed power-saving algorithm does not guarantee the hard deadline, we still try to stop missed deadlines from happening. According to the experimental results shown in Figure 5, there are two settings we can choose, *α* = *D*_*sr*⁡_ × 0.2 and 0.4 when *β* = *D*_*sr*⁡_ − *α*. There was no deadline missed in these two cases. Since the energy consumption of *α* = *D*_*sr*⁡_ × 0.2 is less than *α* = *D*_*sr*⁡_ × 0.4, we chose *α* = *D*_*sr*⁡_ × 0.2 and *β* = *D*_*sr*⁡_ − *α* as the final setting of ED^3^VFS. In this setting, both energy consumption and performance of ED^3^VFS are superior to the original D^3^VFS. Actually, if energy consumption is more important than performance in a given system, setting *α* = *D*_*sr*⁡_ × 0.6 and *β* = *D*_*sr*⁡_ − *α* is also a good choice. In that case, more energy can be saved and a certain level of performance maintained.

**Pseudocode 1:**
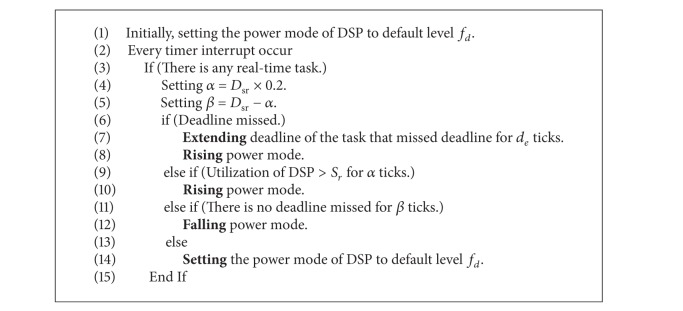
Pseudocode of enhanced deadline-driven dynamic voltage and frequency scaling (ED3VFS).

### 4.3. Two-Level Deadline-Aware Hybrid Load Balancer

For systems limited by battery power, letting all processor cores work continuously in active mode is not a good idea, as much energy will be consumed. In a multicore system, balancing the workload between each core can reduce the completion times of all tasks. Processor cores thus can turn to sleep mode for longer times and save more energy.

**Figure 6:**
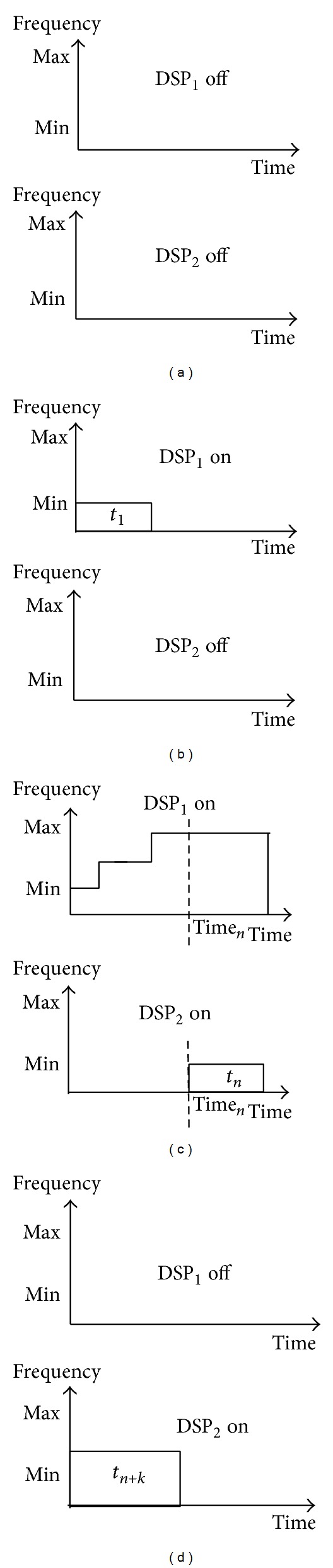
Example of load unbalance. (a) Initial state, (b) Task_1_ released and turning on DSP_1_. (c) Task_*n*_ released while DSP_1_ worked in full speed and turning on DSP_2_. (d) DSP_1_ finished all of tasks that dispatched to it and turning off DSP_1_.

In this paper, a novel task dispatch algorithm, called two-level deadline-aware hybrid load balancer (TLDHLB), is presented. The first level is the load imbalance strategy used for saving static power, and it was inspired by [44]. The basic idea of the first level of task dispatch is dispatching tasks to the processor cores working in active mode and turning off the processor cores when all tasks are finished. For example, suppose there are one MPU and two DSPs in the system. Initially, the system will turn off two DSPs until there are tasks needing to be processed by DSP, as shown in Figure 6(a). When task_1_ was released, MPU will check the state of the DSPs. If there is no DSP working in active mode, then turn on DSP_1_ and dispatch task_1_ to DSP_1_. According to ED^3^VFS, DSP_1_ will work at the default speed, normally, and at the lowest speed in the beginning, as shown in Figure 6(b).  Figure 6(c) shows that if task_*n*_ is released at time_*n*_ while DSP_1_ works at full speed, then turn on DSP_2_ and dispatch task_*n*_ to DSP_2_.  Figure 6(d) shows that, after DSP_1_ finished all tasks assigned to it, it will turn itself off by MEDF.

**Pseudocode 2:**
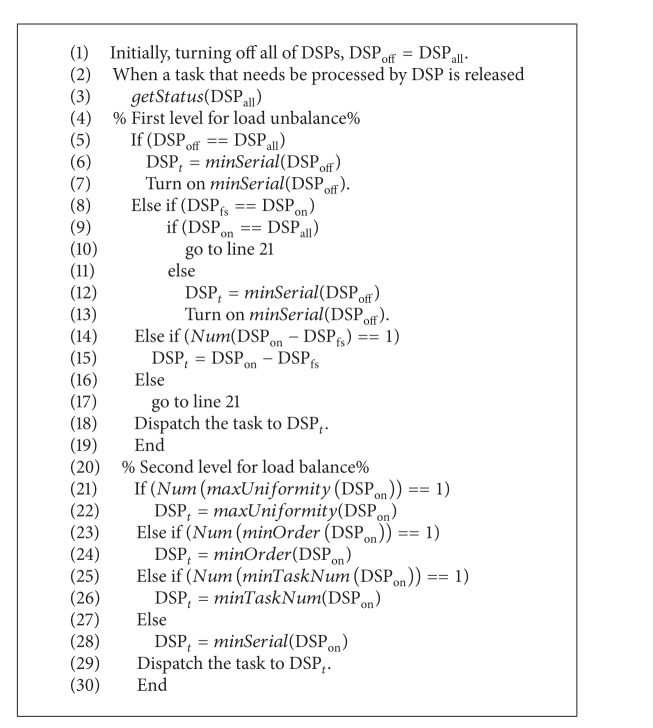
Pseudocode of two levels deadline-aware hybrid load balancer.

The second level is used for load balance. When there are two or more DSPs working in active mode, the load balance strategies in the second level will be used to dispatch tasks that are newly released. Unlike traditional systems that do not contain real-time tasks, there are simultaneously real-time tasks and normal tasks in a system in our assumption. Traditional load balance strategies were not designed for real-time systems, so we propose a new dispatch criterion to process the problem of dispatch in real-time tasks. We also combined other criteria to allow our load balance algorithm to process real-time tasks and normal tasks simultaneously and improve robustness.

**Figure 7:**
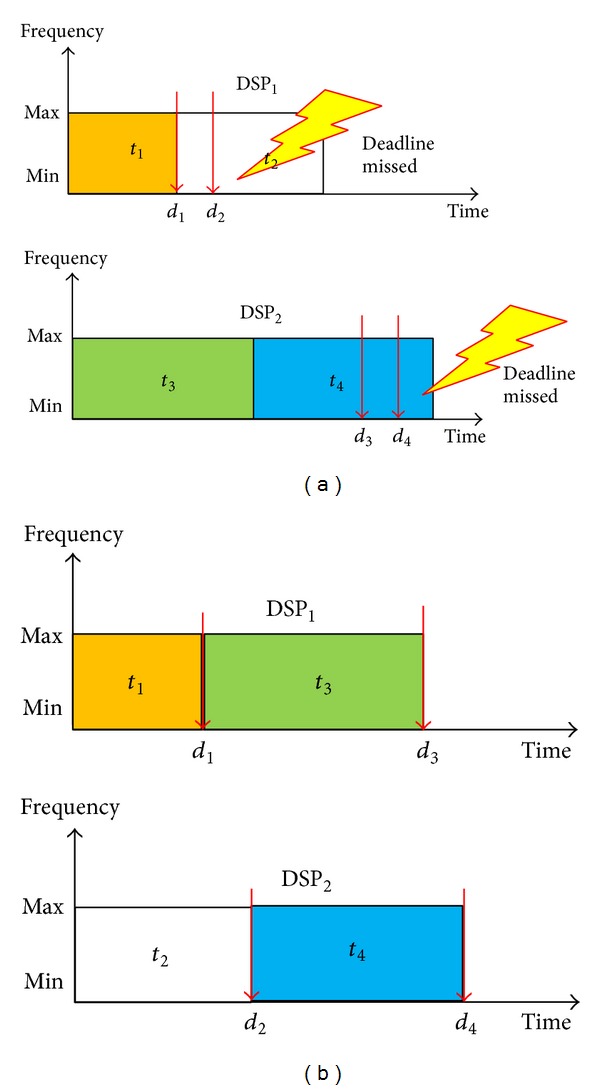
Example of real-time tasks dispatch. (a) Distribution of task deadlines is uneven. (b) Distribution of task deadlines is uniform.

The novel strategy uses the distribution of task deadlines as the criterion for load balance. According to our observation, the more uniform the distribution of task deadlines is, the lower the missing-deadline probability will be.  Figure 7 is a simple example that supports our observation. There are two DSPs and four tasks; Figure 7(a) shows that one of the dispatch results' distribution of task deadlines is uneven. In this example, task_1_ is finished at its deadline, *d*_1_, so that there is not enough time to execute task_2_ and a deadline missed occurs. A similar situation occurred in DSP_2_ for task_4_.  Figure 7(b) shows that a different dispatch result features a more uniform distribution of task deadlines. In this situation, the time slot between two deadlines is longer, which means that there is more time to execute the next task when a task is finished, leading to the probability of a deadline missed being lower. Now, the problem is how to express the distribution of task deadlines.

In this paper, the variance of task deadlines was used as the feature of deadline distribution. Since variance expresses how far a set of numbers is spread out, variance can be used to express the density of data distribution, which is what we need. A smaller variance of task deadlines implies that the time slot between two task deadlines is shorter. Equation (8) is the formula of variance, where *N* is the number of data, *x*_*i*_ expresses *i*th data, and $\overset{-}{x}$ is the data mean:

(8) $$\begin{array}{c} \mathrm{Var}\left(X\right)=\frac{1}{N}\overset{N}{\underset{i=1}{\sum }}{\left(x_{i}-\overset{-}{x}\right)}^{2}. \end{array}$$

Except for the distribution of task deadlines, the other three strategies were combined to dispatch normal tasks. These three strategies can also be used to dispatch real-time tasks when the uniformity of deadline distributions is equal. The first strategy is the execution order of the task. This means that the dispatcher will dispatch a task to the DSP that provides higher priority. For example, assume there are two DSPs and two tasks on each DSP. When a new task is released, if the execution order of the task is second in DSP_1_ and third in DSP_2_, this task will be dispatched to DSP_1_. The second strategy is the number of tasks. The dispatcher will dispatch a task to the DSP that has the fewest number of tasks on it. When the dispatcher cannot make the decision via the three strategies in the second level mentioned above, then the last strategy will be used. The last strategy is very simple, it simply chooses the DSP whose serial number is the minimum and is working in active mode. The pseudocode of two levels deadline-aware hybrid load balancer is shown in Pseudocode 2, where DSP_all_ is the set of all DSPs, DSP_*off*⁡_ is the set of DSPs that are not staying in active mode, DSP_*t*_ is the target that the task will be dispatched to, DSP_*on*⁡_ is the set of DSPs that are staying in active mode, DSP_fs_ is the set of DSPs that are working at full speed, function *getStatus*() is used to obtain the status information of the DSPs, function *minSerial*() returns the DSP whose serial number is the minimum, function *Num*() returns the number of input data, function *maxUniformity*() returns the DSP whose uniformity of deadline distribution is the maximum, function *minOrder*() returns the DSP that can provide the highest priority to new released task and function, *m* *i* *n* *T* *a* *s* *k* *Num*() returns the DSP whose number of tasks is the fewest.

What is worth noticing is that when calculating the uniformity of deadline distribution, we should take the newly released task into account because we want to find a DSP whose deadline distribution is still uniform after inserting the newly released task. Furthermore, *maxUniformity*(), *minOrder*(), and *m* *i* *n* *T* *a* *s* *k* *Num*() may return more than one DSP, when there are two or more DSPs with the same status. In that case, TLDHLB will use another strategy to dispatch the task and is why we combined four criteria to become a hybrid strategy.

## 5. Experiments

In this section, we describe the experimental environment and the setting of parameters. Experimental results and analyses are then shown.

### 5.1. Experimental Environment

**Figure 8:**
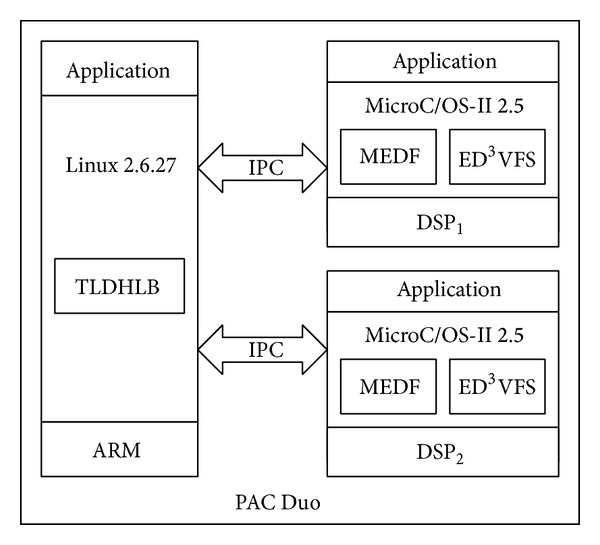
System architecture.

In this study the PAC Duo platform was used for the experiments and includes an ARM926 processor and two PACDSPs. The operating system kernel running on ARM is Linux 2.6.27. We ported the MicroC/OS-II kernel (version 2.5) to the PACDSPs and implemented the proposed power-aware scheduling algorithm on the MicroC/OS-II kernel and the proposed load dispatch algorithm on ARM.  Figure 8 shows the experimental system architecture, while Table 2 shows the operating frequencies used in the experiments and the corresponding voltages. In the experiments, we used a digital multimeter (FLUKE 8846A) to measure the voltage and current of the PACDSPs, the data of which was used to calculate energy consumption.

### 5.2. Experimental Settings

In the experiments, we used matrix multiplication, *π* calculation, quick sort, jpeg decoder, and histogram equalization as the workload. Other than the proposed algorithms, we also implemented two load balance algorithms and three frequency scaling strategies as the comparisons.  Table 3 shows the algorithm usage in the experiments.

- Seven sets of settings were used. The first set is the proposed algorithms and combines the proposed load dispatch algorithm and power-aware scheduling algorithms. Worthy of note is that the DSPs on our experimental platform cannot turn off and then turn on, so we scaled the operating frequency to the lowest frequency to represent the DSP as being turned off and assumed the energy consumption was zero until the proposed algorithms turn it on again.
- The second set to the fourth set used the same load balance algorithm. In these three sets, the load balancer used the number of tasks as the criterion to dispatch tasks. The frequency scaling strategies used in the second and third sets were two static settings: one is the highest operating frequency and the other is the lowest operating frequency. The fourth set used Linux-ondemand [45] as the frequency scaling strategy. Linux-ondemand is a dynamic frequency scaling algorithm; it is used in Linux kernel and dynamically scales the operating frequency according to the utilization of the processor.
- The fifth set to seventh set used the utilization of processor as the criterion to dispatch tasks. The frequency scaling strategies used in these three sets were the highest frequency, the lowest frequency, and Linux-ondemand in ordering. Except for the first set, the other settings use original scheduling used in MicroC/OS-II to schedule tasks.

For each task, we used the times of the average execution time of the task as its deadline, from one time to five times. There are five settings of task deadline for each set of settings in the experiments.

### 5.3. Experimental Results

#### 5.3.1. Comparison of Energy Consumption

**Figure 9:**
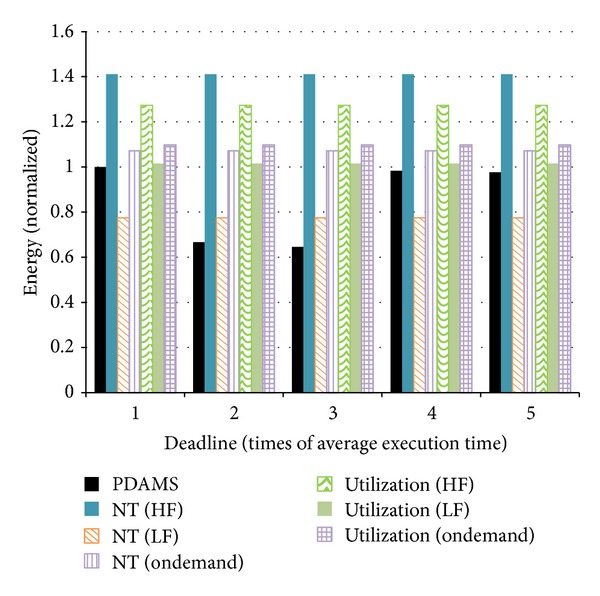
Comparison of energy consumption.

Figure 9 shows the comparison of energy consumption. The vertical axis shows the energy consumption while the horizontal axis shows the setting of the task deadline. The results show that the energy consumption of the proposed algorithms is lower than that of other algorithms in almost every case. Compared with other algorithms, the proposed algorithm can reduce energy consumption by up to 54.2%. Experimental results also show that using the number of tasks as the criterion of load balance and always working in the lowest operating frequency can reduce energy consumption the most. The consequence is obvious and predictable, but the computing capacity under this condition is not satisfying. Although the proposed algorithm considered saving static power, due to hardware constrains, the static power consumption could barely be measured independently. As a result, static power consumption was not taken in count in the experiments. Besides, the overhead of scaling voltage and frequency has not been considered either. These will be added to our next work.

#### 5.3.2. Comparison of Performance

**Figure 10:**
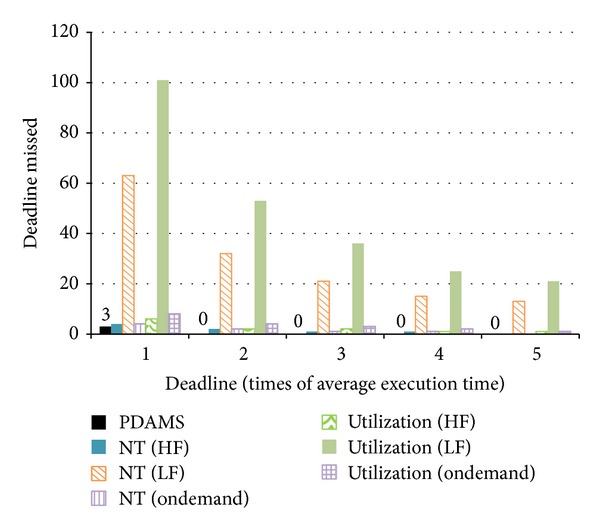
Comparison of performance.

Other than energy consumption, performance is a very important criterion to evaluate the effect of an algorithm. How to deal with the tradeoff between energy and performance is a difficult issue. Differing from traditional systems, the finish time of an overall system cannot represent the performance completely in a real-time system. For a real-time task, there is no difference between a system finishing the task very quickly and finishing the task just at deadline. Before the deadline of a real-time task, no matter what time is required to finish it, the effects of the real-time task are the same. Therefore, the number of missed deadlines is a better criterion to represent the performance of a real-time system.  Figure 10 offers the performance comparisons. The vertical axis shows the number of deadlines missed while the horizontal axis shows the setting of task deadlines. The results show that the performance of the proposed algorithms is the best. Except for setting the deadline of each task into one time of an average execution time, there is no deadline missed while using the proposed algorithms. Since there are only two slave processor cores in our experimental platform, when there are more than three real-time tasks and their deadlines are just one time of their average execution time, missing deadlines cannot be avoided. Worth to note is that even DSPs always run at the fastest frequency, using less appropriate dispatch method and scheduling algorithm may produce more missed deadlines. The proposed algorithm tries to decrease the probability of missing deadline not only in load dispatch, but also in scheduling. That is why the proposed algorithm has the chance to use less energy and get higher performance.

Although always using the lowest operating frequency can reduce energy consumption the most, the performance is not acceptable. The number of deadlines missed is much more than other algorithms, regardless of which load balance algorithm is used. Experimental results show that the proposed algorithms found a good balance point between energy consumption and performance. By considering the deadline of tasks, the distribution of task deadlines is more uniform on each slave processor core. This can reduce the probability of deadlines being missed. A lower probability of deadline missed not only represents higher performance, but also reduces more energy consumption because there is a longer time that the system will work in lower speed when using ED^3^VFS. Moreover, the concept of load imbalance made the proposed algorithms reduce more energy consumption. Differing from dynamic voltage and frequency scaling technology that only reduces the dynamic power, load imbalance also reduces static power.

Both the number of tasks and processor utilization are the most popular strategies to dispatch tasks in real systems, which is why we chose them as the comparisons. The aim of this paper is to develop not only a novel and effective load balance algorithm, but also an algorithm that can be applied in a real environment. Although the performance of some state-of-the-art algorithms may be better than the proposed algorithm, it is very difficult to satisfy their assumptions. Hence, these kinds of algorithms are hard to make work in a real environment. The other reason is the similarity of assumptions that all the algorithms used in this paper do not need the worst case execution time. Nowadays, portable devices that feature the ability to connect to internet are very popular, such as smart phones and tablet PCs. When a user downloads an application from the internet, there is no way for the system to get the worst case execution time of this application immediately. Although not using the worst case execution time of the tasks made the proposed algorithms unable to guarantee the hard-deadline, the proposed algorithm still tries to avoid missing deadline and become more flexible. We implemented the proposed algorithm in a real platform and the experimental results show that the proposed algorithms work well and are superior to others in general performance.

## 6. Conclusion

This paper applied a solution to the problems of load dispatch and power saving in a real-time system on a multicore platform, called power and deadline-aware multicore scheduling. The proposed algorithm simultaneously considers dynamic power, static power, and load balance. To reduce the dynamic power, we implemented MEDF and fine-tuned the parameters of D^3^VFS to save more power and improve performance. The concept of load imbalance was introduced in saving static power. Instead of dispatching the workload to every processor core equally, the proposed algorithm turns power on only in parts of processor cores and lets other unnecessary cores turn to sleep mode or turn-off. Finally, deadline is used as a novel strategy for load balance between processor cores in active mode. Combining load imbalance and load balance, this paper proposed a two-level task dispatch algorithm called two-level deadline-aware hybrid load balancer.

To verify that the proposed algorithms are useful, we implemented them on a multicore platform, PACDuo. We also implemented some load balance algorithms and frequency scaling algorithms for comparison. Experimental results show that compared to six combinations of load balance algorithms and frequency scaling algorithms, the proposed algorithms can reduce energy consumption by up to 54.2% and the performance of the proposed algorithms is superior to others. However, much work still needs to be completed in the future. Some areas for future study include (1) adding theoretical analysis to support the proposed algorithm, (2) modelling the energy consumption more detailed, (3) considering the demands of hard-real-time and task migration while keeping the algorithm light-weight, and (4) introducing the concept of heuristic algorithms and improving the proposed algorithms.

## Figures and Tables

**Table 1:** Overall settings of *α* and *β*.

Settings	Group 1 (*α* = *β*)		Group 2 (*α* = *D*_*sr*⁡_ − *β*)	
	*α*	*β*	*α*	*β*
1	0.2*D*_*sr*⁡_	0.2*D*_*sr*⁡_	0.2*D*_*sr*⁡_	0.8*D*_*sr*⁡_
2	0.4*D*_*sr*⁡_	0.4*D*_*sr*⁡_	0.4*D*_*sr*⁡_	0.6*D*_*sr*⁡_
3	0.6*D*_*sr*⁡_	0.6*D*_*sr*⁡_	0.6*D*_*sr*⁡_	0.4*D*_*sr*⁡_
4	0.8*D*_*sr*⁡_	0.8*D*_*sr*⁡_	0.8*D*_*sr*⁡_	0.2*D*_*sr*⁡_

**Table 2:** Operating voltage and frequency of PACDSP.

Power mode (operating mode)	Voltage (V)	Frequency (MHz)
7	1.0	204
6	1.0	136
5	0.9	102
4	0.9	68
3	0.9	51
2	0.8	34
1	0.8	24

**Table 3:** Usage of algorithms for experiments.

Set	Load dispatch			Frequency scaling			
	TLDHLB	The number of tasks	Utilization	ED^3^VFS + MEDF	Always in the highest frequency	Always in the lowest frequency	Linux-ondemand
1 PDAMS	*✓*			*✓*			

2 NT (HF)		*✓*			*✓*		

3 NT (LF)		*✓*				*✓*	

4 NT (Ondemand)		*✓*					*✓*

5 Utilization (HF)			*✓*		*✓*		

6 Utilization (LF)			*✓*			*✓*	

7 Utilization (Ondemand)			*✓*				*✓*
